# Perfumes

**Published:** 1858-09

**Authors:** 


PERFUMES.—Add to one pint of *spirits of wine*, that is, best alcohol, one ounce bergamot: *or*, one ounce otto of santal: *or*, otto of cloves, one drachm, with half an ounce each of bergamot and French lavender: *or*, half an ounce essence of lemons, with a quarter of an ounce of otto of lemon-grass: *or*, half an ounce of otto of orange-peel, with a quarter of an ounce of otto of petit-grain. Thus, four delightful and cheap perfumes are made, says *The Scientific American.*

